# Amelioration effect of *Lactobacillus plantarum KFY02 on* low-fiber diet-induced constipation in mice by regulating gut microbiota

**DOI:** 10.3389/fnut.2022.938869

**Published:** 2022-08-24

**Authors:** Ruokun Yi, Xin Zhou, Tongji Liu, Rui Xue, Zhennai Yang

**Affiliations:** ^1^Beijing Advanced Innovation Center for Food Nutrition and Human Health, Beijing Engineering and Technology Research Center of Food Additives, Beijing Technology and Business University, Beijing, China; ^2^Department of Cardiology, First Affiliated Hospital, Chongqing Institute of Interventional Cardiology, Army Medical University (Third Military Medical University), Chongqing, China

**Keywords:** *Lactobacillus plantarum*, low-fiber diet, constipation, mice, gut microbiota

## Abstract

This study aimed to examine the ameliorating effect of *Lactobacillus plantarum* (LP) KFY02 on low-fiber diet-induced constipation in mice. LP-KFY02 was isolated from the natural fermented yogurt in Korla of Xinjiang. The mice with low-fiber diet-induced constipation in experimental groups were administered 1 × 10^9^ CFU/kg LP-KFY02 (KFY02H) and 1 × 10^8^ CFU/kg LP-KFY02 (KFY02L). After LP-KFY02 treatment with constipation mice, the mice fecal water content, intestinal transit ability and defecation time of constipated mice were improved. The mice fecal flora diversity, abundance and structure of the intestinal flora were regulated to the balanced state. The mice serum levels of gut motility related neuroendocrine factors have been increased, the intestinal mucosal barrier function and gut motility related gene expression were regulated in mice colon tissues. At the same time, the mice colon tissue damage were improved. These parameters in the KFY02H group were close to the normal group. These results suggested that LP-KFY02 could be considered as a potential probiotic to help alleviate low-fiber diet-induced constipation. They also provided a theoretical basis for the study of probiotics to relieve constipation by regulating intestinal flora.

## Introduction

Constipation is a high-incidence disease worldwide, the increasing global prevalence of the symptoms of constipation adversely affects the quality of life of symptomatic patients (1). With the acceleration of people’s life rhythm, the changes in dietary structure and habits, the influence of social and physiological factors, the incidence of constipation continues to increase every year. Insufficient dietary fiber in the diet is one of the leading causes of constipation (2). Constipation is divided into functional constipation (FC), irritable bowel syndrome with constipation, opioid-induced constipation, and functional defecation disorder. Among these, the prevalence of FC is the highest (3).

FC is intractable constipation characterized by colonic dysfunction and conduction abnormalities resulting in weakened intestinal transit capacity. It can even cause a series of serious complications (4). The research on the pathogenesis and mechanism of FC has gradually increased in recent years, including abnormalities in brain–gut peptides, intestinal flora, interstitial cells of Cajal, and mental factors (5).

The traditional treatment of constipation is mainly use osmotic and secretory laxatives, this method is often unable to effectively cure constipation, the abuse of laxatives will damage the colon nervous system and make constipation more serious (6). The mechanism of intestinal motility changes based on the interaction between the gut microbiota and the enteroendocrine system, and the intestinal immune system has received extensive attention (7). A large number of studies have shown that gut microbiota plays a key role in gut health. The microbiota affects various pathophysiological activities of the host. Establishing and maintaining a beneficial balance between the gut microbiota and the human body is necessary for normal bowel function (8). At the same time, studies have shown that the distribution of intestinal flora in low fiber diet-induced constipation patients is significantly different from that in the normal population, mainly manifested in the decrease in the abundance of common beneficial bacteria (*Bifidobacterium*, *Lactobacillus*, and so on) and the increase in the abundance of potential pathogenic bacteria (*Enterococcus*, *Enterobacteriaceae*, and so on) (9). It is clinically recommended to improve the composition of intestinal flora using probiotics to relieve constipation (10). The main mechanisms are as follows: (1) Probiotics help to regulate the balance of intestinal flora. The intestinal flora can produce a series of metabolites, including bile acids, short-chain fatty acids, hydrogen sulfide, and methane. These products affect intestinal motility by acting on the intestinal wall (11). (2) The gut microbiota is involved in the production of neuroendocrine factors and gastrointestinal hormones, including motilin (MTL), substance P (SP), gastrin (GAS), vasoactive intestinal peptide (VIP), and 5-hydroxytryptamine (5-HT). Neuroendocrine factors work with the nervous system to regulate the movement, secretion and absorption of digestive organs (12). At the same time, the gut microbiota also interacts with the enteric nervous system and enteric neurotransmitters. Among them, glial cell line–derived neurotrophic factor (GDNF) has an important effect on the innervation of colonic myometrium and the function of ganglion cells (13). Transient receptor potential vanilloid 1 (TRPV1) can regulate intestinal motility by mediating 5-HT release (14). (3) The intestinal mucosa is exposed to a large number of antigens and gut microbiota, and intestinal immune homeostasis depends on the synergistic interaction of gut microbiota, intestinal epithelial cells and gut-associated lymphoid tissue (GALT). Among them, the tight junction protein plays a key role in the normal function of the intestinal mucosal barrier, including claudin and occluding (15). C-kit is a specific marker of interstitial cells of Cajal (ICC), and stem cell factor (SCF) is the ligand of c-kit, the decreased expression of the them will have a certain impact on the growth and development of ICC, which is closely related to gastrointestinal diseases (16). At the same time, mediators released by colonic immune cells regulate various digestive functions. Therefore, the disturbance of the colonic immune system can lead to the abnormal reaction of intestinal contents, and this mechanism is also considered to be an important cause of colonic motility disorders (17). Therefore, improving constipation via changing the composition of gut microbes by probiotics is one of the effective ways. However, the development of safe and effective probiotics is still an urgent issue to be addressed.

The Xinjiang Uygur Autonomous Region is located in the frontier area of southwestern China, and the livestock industry is the local pillar industry. Many ethnic minorities reside in Xinjiang, and most of them have the habit of drinking dairy products. Their homemade natural fermented yogurt is one of the most common dairy products (18). Natural fermented foods mostly use natural inoculation. The fermentation process is a relatively open system, involving a wide variety of microorganisms. The dynamic changes in microorganisms are also very complex. A relatively stable flora and ecological environment have been gradually formed, and the selection has retained abundant and excellent microbial resources during the long-term fermentation and domestication process (19).

Therefore, this study explored the improvement effect of lactic acid bacteria isolated from Xinjiang natural fermented yogurt on a low-fiber diet-induced constipation model. Based on the findings of this study, we will explore safe and effective microbial resources that can improve constipation and provide research data support for methods to improve FC by regulating intestinal flora disturbance.

## Materials and methods

### Experimental strain

The experimental strain *Lactobacillus plantarum* (LP) KFY02 was isolated from the natural fermented yogurt in Korla of Xinjiang Uygur Autonomous Region. The strain was preserved at the China General Microbiological Culture Collection Center (CGMCC, Beijing, China) with a preservation number CGMCC no. 15638. *Lactobacillus delbrueckii* subsp. *Bulgaricus* NQ2508 (LB) was obtained from the China Center of Industrial Culture Collection (preservation number: CICC 6047).

### Animal experiment design

**FIGURE 1:**
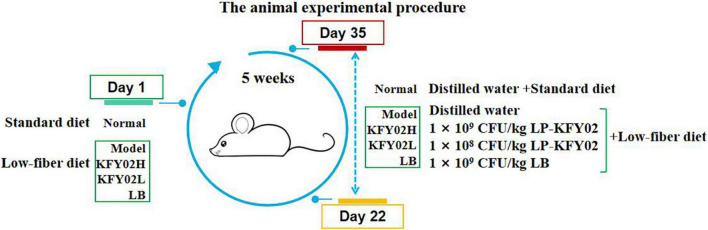
Implementation process of animal experiment in this study.

Fifty 6-week-old male Institute of Cancer Research (ICR) mice were obtained from the Chongqing Medical University, Chongqing, China [SCXK (YU) 2017-0001]. The mice were raised in a climate-controlled room (temperature 25 ± 2°C, relative humidity 50 ± 5%) on a 12 h light/dark cycle (lights on at 7 a.m.) with *ad libitum* access to standard chow and water. After 7 days of adaptive feeding, the mice were randomly and equally divided into five groups: normal, model, positive control, KFY02H group: high-dose LP-KFY02, KFY02L group: low-dose LP-KFY02, with 10 mice per group (five mice per cage). The experimental procedure is shown in Figure 1. The mice in the normal group were fed a standard diet. The other groups were fed a low-fiber diet containing 41.5% corn starch, 24.5% milk casein, 10.0% sucrose, 10.0% dextrin, 7.0% mineral mixture, 6.0% corn oil, and 1.0% vitamin mixture. The experimental period was 5 weeks. In the normal group, a standard diet was given continuously for 5 weeks. From the 22nd day, the mice were dosed daily by gavage with distilled water for 14 days. In the model group, a low-fiber diet was continuously given for 5 weeks. From the 22nd day, the mice were given distilled water by gavage each day for 14 days. In the experimental group, a low-fiber diet was continuously given during the experimental period. From the 22nd day, the mice in the KFY02H group were administered 1 × 10^9^ CFU/kg LP-KFY02, the mice in the KFY02L group were administered 1 × 10^8^ CFU/kg LP-KFY02, and the mice in the positive control group were administered 1 × 10^9^ CFU/kg LB per day for 14 days. During the experiment, the mice body weight were recorded per week.

On the 35th day, all mice were fasted for 16 h and then given activated carbon solution (10 mL/kg BW). In each group, five mice were observed to record the time of the first black stool, and the remaining five mice were used to measure the gastrointestinal transit rate. The gastrointestinal transit rate of activated charcoal was calculated according to the following equation:

Gastrointestinal transit rate (%) = (activated carbon pushes the distance in the small intestine/total length of small intestine) × 100%

For measuring mice fecal water content, the fresh mice stools were collected and weighed on 22nd and 35th day, and the dry weight was obtained by drying fresh stools in an oven at 70°C for 48 h. The mice fecal water content was calculated according to the following equation:

Fecal water content (%) = [(fresh feces weight - dry feces weight)/fresh feces weight] × 100%

For microbial analysis, the fresh mice feces were collected in individual sterile eppendorf tubes on 35th day, then stored at –80°C for further deoxyribonucleic acid (DNA) extraction.

At the end of the experiment, the mice were sacrificed by cervical dislocation. The blood from the mice were centrifuged at 4°C and 4,000 rpm for 10 min to collect the serum. The colon tissues were collected and stored at –80°C for further experiments.

### Deoxyribonucleic acid extraction, polymerase chain reaction amplification, and sequencing

Genomic DNA of mice feces were extracted using a FastPrep DNA extraction kit (QBIOGENE, United States), and the extracted genomic DNA was detected by 1% agarose gel electrophoresis. The polymerase chain reaction (PCR) amplification and sequencing region was 338F_806R, and the amplification primers used universal primers (338F/806R) of the 16S rRNA gene V3–V4 region. The primer sequences were 338F (5′-ACTCCTACGGGAGGCAGCAG-3′) and 806R (5′-GGACTACHVGGGTWTCTAAT-3′). PCR formal experiments used a TransGen AP221-02: TransStart Fastpfu DNA Polymerase, 20-μL reaction system. Reaction conditions: 95°C, 10 min, 95°C, 15 s, 58°C, 30 s, 72°C, 30 s, 40 cycles. DNA extraction, PCR amplification, and sequencing were done in collaboration with Shanghai Majorbio Bio-pharm Technology Co., Ltd. The 16S rRNA sequence data were analyzed using the QIIME2-pipeline on the Majorbio Cloud Platform.^1^

### Detection of the serum levels of motilin, substance P, gastrin, vasoactive intestinal peptide, and 5-hydroxytryptamine

The serum levels of MTL, SP, gastrin, VIP, and 5-HT were quantified using ELISA kits following the manufacturer’s protocols (Shanghai Meilian Biotechnology Company, Shanghai, China).

### Histological evaluation

A portion of mice colon tissues were cut and immersed in 10% formalin solution. Paraffin-embedded sections were made and stained with hematoxylin and eosin in collaboration with Wuhan Servicebio Technology Co., Ltd. The histological sections were observed under a biological microscope (BX43; Olympus, Japan).

### Quantitative polymerase chain reaction

Total RNA was extracted from colon tissues, and the RNA concentration and purity were determined. According to the instructions of a Revert Aid First Strand cDNA synthesis kit (Thermo Fisher Scientific, Inc., United States), the RNA was reverse transcribed into cDNA and then amplified using a Real-Time PCR System (Step One Plus Real-Time PCR System, Thermo Fisher Scientific, Inc.). The relative mRNA expression of each target gene SCF, c-kit, transient receptor potential vanilloid 1 (TRPV1), glial cell line–derived neurotrophic factor (GDNF), claudin-1, and occludin were calculated using the formula 2^–ΔΔ*CT*^ method with GAPDH as the internal reference gene (Thermo Fisher Scientific, Waltham, MA, United States; Table 1).

**TABLE 1:** Primer sequences used in this study.

Gene name	Sequence
SCF	Forward: 5′-TCAGGGACTACGCTGCGAAAG-3′
	Reverse: 5′-AAGAGCTGGCAGACCGACTCA-3′
c-kit	Forward: 5′-CATAGCCCAGGTAAAGCACAAT-3′
	Reverse: 5′-GAACACTCCAGAATCGTCAACTC-3′
TRPV1	Forward: 5′-AGCGAGTTCAAAGACCCAGA-3′
	Reverse: 5′-TTCTCCACCAAGAGGGTCAC-3′
GDNF	Forward: 5′-TTTTATTCAAGCCACCATC-3′
	Reverse: 5′-AGCCCAAACCCAAGTCA-3′
Claudin-1	Forward: 5′-TTCTGGGAGGTGTCCTACTT-3′
	Reverse: 5′-AGGTGTTGGCTTGGGATAAG-3′
Occludin	Forward: 5′- TAGTGTAGGCTACCCTTATGGAG –3′
	Reverse: 5′-ATGCCCAGGATAGCACTCAC-3′
GAPDH	Forward: 5′-AGGTCGGTGTGAACGGATTTG-3′
	Reverse: 5′-GGGGTCGTTGATGGCAACA-3′

## Statistical analysis

Data were presented as the mean ± standard deviation. The SPSS 19.0 software (SPSS Institute Inc., IL, United States) was used to conduct the analysis. Differences between the mean values for individual groups were assessed with a one-way analysis of variance and Duncan’s multiple range tests. A *p* < 0.05 indicated a statistically significant difference.

## Results

### Body weight changes of mice

**FIGURE 2:**
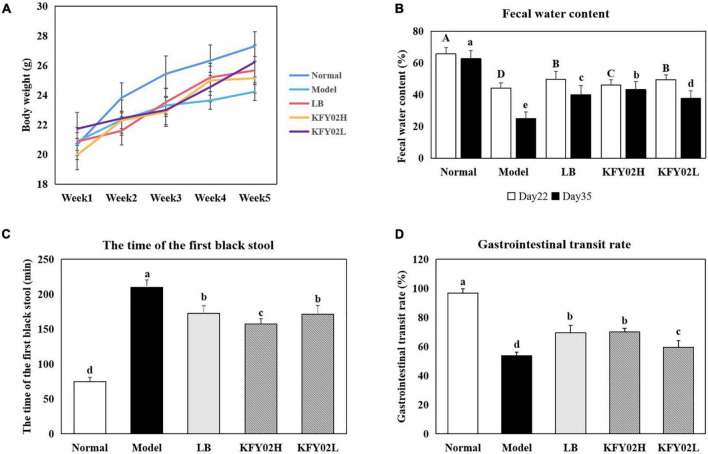
Effect of LP-KFY02 on constipation in mice. **(A)** Body weight; **(B)** fecal water content; **(C)** the time of the first black stool; **(D)** gastrointestinal transit rate. Data are means ± standard deviations. ^a– e^Different superscript letters within a column correspond to significant differences (*p* < 0.05). LB: mice administered with 1 × 10^9^ CFU/kg LB, KFY02H: mice administered with 1 × 10^9^ CFU/kg LP-KFY02, KFY02L: mice administered with 1 × 10^8^ CFU/kg LP-KFY02.

In order to evaluate the effect of LP-KFY02 on the body weight change of constipated mice, the mice body weight were measured per week (Figure 2A). The body weight of mice in each group gradually increased during the experimental period. However, the body weight of low-fiber diet-induced constipated mice were significantly lower than the normal mice (*p* < 0.05). Among them, the body weight of mice in the model group was significantly lower than the KFY02H group, KFY02L group, and the LB group (*p* < 0.05).

### Fecal parameters of mice

The fecal water content can evaluate whether the mice constipation model is successfully induced. Dry stools are common in constipated patients. An increase in fecal water content indicates an increase in intestinal peristalsis, thereby relieving the symptoms of constipation. As shown in Figure 2B, on 22nd day of constipation induction, the fecal water content of mice in the model group, LB group and KFY02H group, KFY02L group decreased to varying degrees, which was significantly lower than the normal group (*p* < 0.05), indicating that the constipation model was successfully established. On 35th day of constipation induction, after gavage with LP-KFY02, the fecal water content of mice increased significantly compared with the model group (*p* < 0.05).

The excretion time of the first black stool in mice is also one of the important indicators for judging the intestinal peristalsis ability of constipated mice. Excessive time to first black stool indicates severe constipation symptoms. As shown in Figure 2C, the excretion time of the first black stool in the normal group, model group, LB group, KFY02H group, and KFY02L group were 74.8 ± 6.26, 209.8 ± 10.62, 172.6.17 ± 10.74, 157.2 ± 7.92, and 171.4 ± 12.50 min, respectively. The time of the first black stool in the KFY02H group was significantly less than the model group, which was closer to the normal group compared with the LB group and KFY02L group (*p* < 0.05).

### Gastrointestinal transit ability of mice

The gastrointestinal transit rate can directly reflect the intestinal motility. In Figure 2D, the gastrointestinal transit rate in the normal group, model group, LB group, KFY02H group, and KFY02L group were 96.73 ± 2.97, 53.70 ± 2.53, 69.52 ± 5.18, 70.05 ± 2.46, and 59.65 ± 4.33%, respectively. The gastrointestinal transit rate of the KFY02H group, KFY02L group significantly increased compared with the model group (*p* < 0.05), KFY02H group was close to the LB group, which indicating that LP-KFY02 can promote intestinal motility.

### Effect of *Lactobacillus plantarum*-KFY02 on the microbial diversity in mice feces

The alpha diversity in mice feces was shown in Table 2. Among these, the observed species index (sobs index) and chao index show community richness, the shannon index and simpson index show bacterial diversity, the coverage data shows community coverage. The normal group, the groups treated with LP-KFY02 and LB had higher community richness and bacterial diversity significantly compared with the model group (*p* < 0.05). At the same time, the sobs index, chao index, and shannon index were significantly higher in the KFY02H group compared with the other groups (*p* < 0.05). The simpson index of KFY02H group was 0.04 ± 0.01, which was significantly decreased compared with the other groups (*p* < 0.05). The alpha diversity results showed the higher community richness and bacterial diversity of constipated mice feces in the KFY02H group.

**TABLE 2:** The alpha diversity in mice feces.

Group	Sobs	Chao	Shannon	Simpson	Coverage
Normal	716.86 ± 224.52^a^	752.06 ± 235.47^a^	4.09 ± 1.20^b^	0.10 ± 0.18^b^	0.99926
Model	111.57 ± 55.54^d^	116.15 ± 57.31^d^	1.74 ± 0.36^e^	0.30 ± 0.10^a^	0.99989
LB	503.71 ± 192.42^b^	516.65 ± 198.97^b^	3.60 ± 1.02^c^	0.10 ± 0.09^b^	0.99966
KFY02H	720.86 ± 131.44^a^	754.89 ± 135.11^a^	4.38 ± 0.29^a^	0.04 ± 0.01^c^	0.99930
KFY02L	304.86 ± 196.94^c^	313.99 ± 197.63^c^	2.29 ± 1.20^d^	0.31 ± 0.24^a^	0.99972

Data are means ± standard deviations.

^a–e^Different superscript letters within a column correspond to significant differences (p < 0.05). LB: mice administered with 1 × 10^9^ CFU/kg LB, KFY02H: mice administered with 1 × 10^9^ CFU/kg LP-KFY02, KFY02L: mice administered with 1 × 10^8^ CFU/kg LP-KFY02.

**FIGURE 3:**
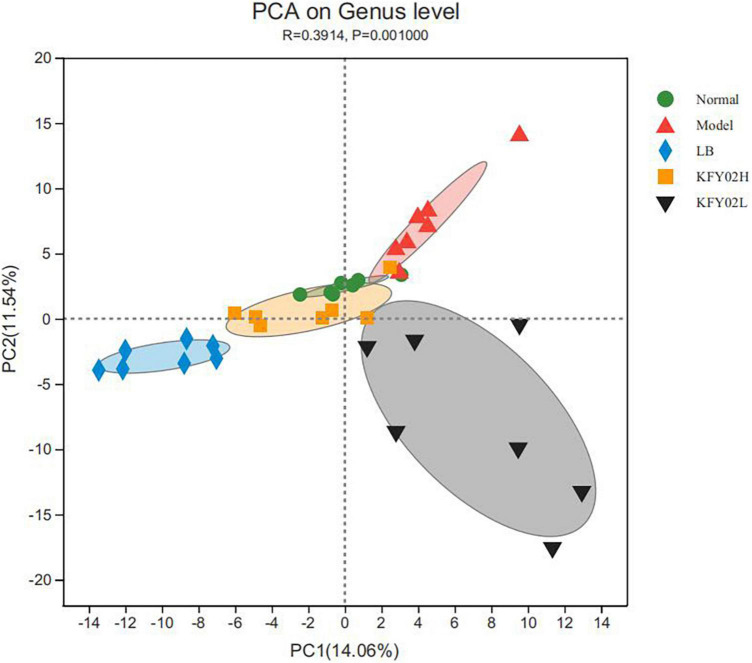
Principal component analysis (PCA) of mice feces. Each colored symbol represents the composition of fecal microbiota of one mice. LB: mice administered with 1 × 10^9^ CFU/kg LB, KFY02H: mice administered with 1 × 10^9^ CFU/kg LP-KFY02, KFY02L: mice administered with 1 × 10^8^ CFU/kg LP-KFY02.

The beta diversity was determined *via* principal component analysis (PCA). In Figure 3, the cluster of the KFY02H group was similar to the normal group but relatively separated from the other groups, indicating that the diversity of gut microbiota in constipated mice reverted to normal after high-dose LP-KFY02 treatment. These results indicated that LP-KFY02 changed the feces microbiota, the fecal flora diversity and abundance in the KFY02H group was similar to the normal group.

### Effect of *Lactobacillus plantarum*-KFY02 on the relative abundance of microbiota in mice feces

**FIGURE 4:**
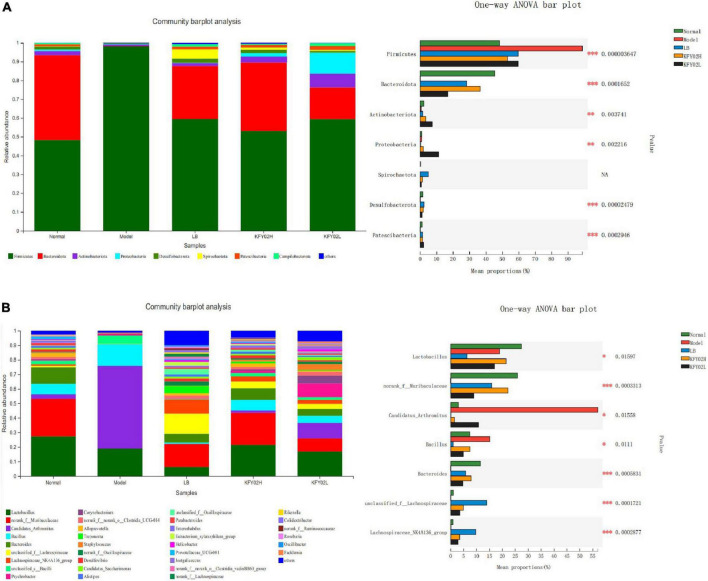
Effect of LP-KFY02 on gut microbiota of constipation mice. **(A)** Mice feces bacterial communities at the phylum level and statistical analysis; **(B)** mice feces bacterial communities at the genus level and statistical analysis; *0.01 < *p* ≤ 0.05, ^**^0.001 < *p* ≤ 0.01, ^***^p ≤ 0.001; LB: mice administered with 1 × 10^9^ CFU/kg LB, KFY02H: mice administered with 1 × 10^9^ CFU/kg LP-KFY02, KFY02L: mice administered with 1 × 10^8^ CFU/kg LP-KFY02.

The composition of the gut microbiota flora at the phyla level and genus level were explored. As shown in Figure 4A, the most dominant phyla in the five groups were *Firmicutes* and *Bacteroidetes*, followed by *Actinobacteria, Proteobacteria*, *Desulfobacterota, Spirochaetota, Deferribacteres, Patescibacteria*, and *Campilobacterota*. Following the statistical results, in the model group, the *Firmicutes* abundance increased significantly, the *Bacteroidetes* abundance decreased significantly compared with the other groups (*p* < 0.05). However, in KFY02H group, the *Firmicutes* abundance decreased significantly, the *Bacteroidetes* abundance increased significantly compared with the model group, the LB group and the KFY02L group (*p* < 0.05), which is close to the normal group.

The composition of the gut microbiota flora at the genus level in the five groups is shown in Figure 4B. Compared with the model group, the *Lactobacillus*, *norank_f_Muribaculaceae*, and *Bacteroidetes* abundance significantly increased in the KFY02H group (*p* < 0.05), which is close to the normal group. These results indicated that composition of the gut microbiota flora in the KFY02H group was similar to the normal group. Therefore, LP-KFY02 maintained the gut microbiota of mice to a balanced state.

### Effect of *Lactobacillus plantarum*-KFY02 on mice serum levels of neuroendocrine factors and gastrointestinal hormones

As shown in Table 3, the levels of gastrointestinal motility–related neurotransmitter MTL, SP, GAS, VIP, and 5-HT significantly decreased in the model group (*p* < 0.05). After treatment with LP-KFY02 and LB, the serum levels of MTL, SP, GAS, VIP, and 5-HT increased significantly, which were close to the level in the normal group (*p* < 0.05). The levels in KFY02H group were the closest to those in the normal group.

**TABLE 3:** Serum levels of MTL, SP, GAS, VIP, and 5-HT in mice.

Group	MTL (pg/mL)	SP (pg/mL)	GAS (pg/mL)	VIP (pg/mL)	5-HT (pg/mL)
Normal	55.83 ± 0.94^a^	79.33 ± 2.59^a^	81.24 ± 4.09^a^	22.82 ± 1.72^a^	21.14 ± 0.83^a^
Model	28.01 ± 0.74^e^	24.57 ± 0.42^e^	24.37 ± 1.88^e^	6.53 ± 1.21^d^	10.63 ± 1.96^e^
LB	49.03 ± 0.99^b^	69.08 ± 3.76^c^	69.87 ± 3.38^b^	16.31 ± 1.79^b^	17.70 ± 0.62^c^
KFY02H	44.60 ± 0.43^c^	71.61 ± 1.72^b^	62.83 ± 2.48^c^	16.49 ± 1.18^b^	18.69 ± 1.01^b^
KFY02L	33.74 ± 0.60^d^	53.38 ± 0.70^d^	47.12 ± 2.48^d^	10.57 ± 0.24^b^	14.18 ± 0.47^d^

Data are means ± standard deviations.

^a–e^Different superscript letters within a column correspond to significant differences (p < 0.05). LB: mice administered with 1 × 10^9^ CFU/kg LB, KFY02H: mice administered with 1 × 10^9^ CFU/kg LP-KFY02, KFY02L: mice administered with 1 × 10^8^ CFU/kg LP-KFY02.

### Histological evaluation of the mice colon tissues

**FIGURE 5:**
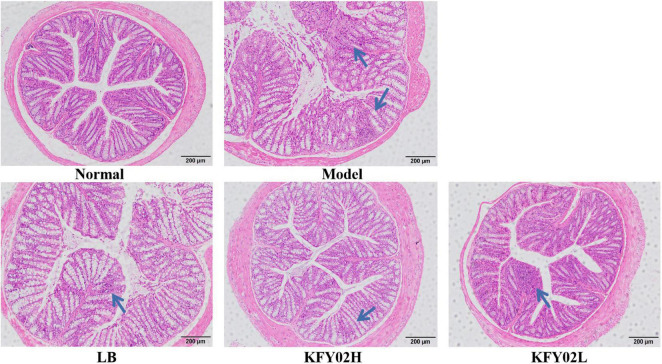
Pathological assessment of H&E-stained mice colon sections; magnification 200×.

As shown in Figure 5, the mice colonic structure was intact and the mucosal glands were arranged regularly in the normal group. However, the mice mucosal pathology showed obvious rupture of the muscle layer, destruction of the colon structure, and infiltration of inflammatory cells in the model group. After gavage with LP-KFY02 and LB, the pathological changes and the infiltration of inflammatory cells in the mice colon tissues improved. According to the histological observation, the recovery effect in the KFY02H group on colon tissue was better than that in the KFY02L group, which was close to that in the normal group.

### mRNA expression levels of stem cell factor, c-kit, transient receptor potential vanilloid 1, glial cell line–derived neurotrophic factor, claudin-1, and occludin in the mice colon tissues

**FIGURE 6:**
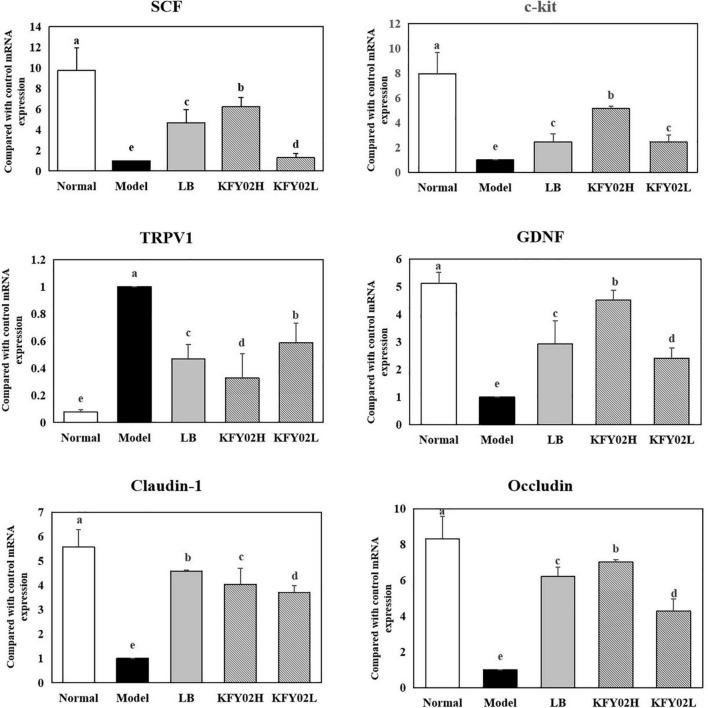
mRNA expression levels of SCF, c-kit, TRPV1, GDNF, claudin-1, and occludin in the mice colon tissues. Data are means ± standard deviations. ^a– e^Different superscript letters within a column correspond to significant differences (*p* < 0.05). LB: mice administered with 1 × 109 CFU/kg LB, KFY02H: mice administered with 1 × 109 CFU/kg LP-KFY02, KFY02L: mice administered with 1 × 108 CFU/kg LP-KFY02.

We also evaluated the mRNA expression levels of SCF, c-kit, TRPV1, GDNF, claudin-1, and occludin. Figure 6 shows that the expression of SCF, c-Kit, GDNF, claudin-1, and occludin was upregulated in the colon tissue of mice with constipation (*p* < 0.05). In contrast, the expression levels of these markers were downregulated in the model group. Also, the expression of TRPV-1 was downregulated in the KFY02H group, KFY02L group compared with the model group (*p* < 0.05). The mRNA expression levels in the KFY02H group was similar to that in the normal group.

## Discussion

The pathogenesis of FC is mainly due to abnormal defecation dynamics, decreased intestinal smooth muscle tension and weakened peristalsis, mechanical obstruction of intestinal peristalsis, and insufficient intake of water and cellulose (20). The lack of dietary fiber and insufficient water in the diet are some of the most important factors for constipation (2). The continuous exploration of the pathophysiological mechanism of FC and the in-depth study of the intestinal microecology showed the imbalance of intestinal flora as one of the pathogeneses of FC (21). Clinical studies have shown that a low-fiber diet destroys the gut microbiota, and improving the intestinal flora through the intake of probiotics is one of the important ways to relieve FC (22). Many studies suggested that specific probiotics helped reduce bowel transit time. Recent research found that *Bifidobacterium bifidum* G9-1 (23) and *Lactobacillus rhamnosus* strains relieved loperamide-induced constipation (24). At the same time, the effect of *Bifidobacterium animalis* subsp. *lactis* MN-Gup on the alleviation of constipation in BALB/c mice and humans have been determined (25). The mechanism of probiotics in the treatment of FC has the following three points:

1. Probiotics can regulate intestinal flora in a beneficial direction. Probiotic metabolites also affect gut motility.

The composition of gut microbiota was significantly different in patients with FC compared with healthy people in terms of quantity and type. Studies showed that the application of probiotics to patients with FC could directly supplement the insufficiency of their intestinal beneficial bacteria and improve the problem of intestinal flora imbalance (26).

At the same time, the products of bacterial fermentation in the gut also affect gut motility (27). Probiotics can produce various organic acids such as short-chain fatty acids and bile acids through metabolism (8). The organic acids act on the intestinal wall to reduce the pH in the intestinal tract and regulate gastrointestinal motility and gastrointestinal function (28). Among these, *Lactobacillus* can decompose oligosaccharides in the intestinal tract; produce organic acids such as butyric acid, acetic acid, propionic acid, and so forth; improve intestinal peristalsis; reduce defecation time; and produce a large amount of volatile fatty acids, which can inhibit the excess of aerobic harmful bacteria, thereby regulating the balance of intestinal flora (29). Other products of gut microbiota fermentation include methane and hydrogen. Methane, as a neuromuscular transmitter, also affects gut motility (26). In addition, chemotactic peptides produced by gut flora, such as formyl-methionyl-leucyl-phenylalanine, stimulate the enteric nervous system, and primary afferents (30).

In our study, after LP-KFY02 treatment, fecal water content, intestinal transit ability and defecation time of constipated mice were improved, the structure of the intestinal flora had also been improved. In particular the mice fecal flora diversity and abundance in the KFY02H group were similar to the normal group. At the same time, the results indicated that composition of the gut microbiota flora in the KFY02H group was similar to the normal group. Therefore, LP-KFY02 maintained the gut microbiota of mice to a balanced state. We considered that favorable intestinal flora environment mediated by LP-KFY02 can relieve constipation by improving intestinal motility, increasing fecal water content, and enhancing intestinal transit ability.

2. Gut microbiota can influence the production of neuroendocrine factors and gastrointestinal hormones, thereby regulating intestinal motility.

Many studies showed that the brain–gut axis was considered to be a key way of interconnecting the nervous system and the gut, and played an important role in the pathogenesis of FC (31). The digestive system is innervated by connections to the central nervous system and the enteric nervous system within the walls of the gastrointestinal tract, which work in concert with the central nervous system reflex and command centers and the neural pathways that control digestive function through the sympathetic ganglia (32).

Enteroendocrine cells (EECs) are specialized epithelial cells distributed in the gastrointestinal mucosal cells. Although their total number is less than 1% of the total intestinal epithelial cells, it is an important carrier to maintain the normal function of the brain–gut axis, and the polypeptide secreted by EEC is a key intermediate transmitter (33). These polypeptides have dual functions of neurotransmitter conduction and hormone secretion. Their main role is to regulate gastrointestinal motility and secretion by transmitting information between central nervous system, enteric nervous system, and gastrointestinal effector cells, thereby realizing the interaction between the brain and the gut (34). The polypeptides mainly include MTL (MTL can contract the gastric body and antrum, relax the pylorus, and play a major role in promoting gastric emptying during the interdigestive period), GAS (GAS can be increased by mechanical or chemical stimulation, thereby increasing sphincter tone and promoting gastrointestinal motility), SP (the main role of SP is to strengthen intestinal smooth muscle contraction, peristalsis, and gastric emptying, and it is the main excitatory neurotransmitter in the regulation of gastrointestinal motility) (35), VIP (VIP is both a gastrointestinal hormone and an inhibitory neurotransmitter, which decreases smooth muscle diastole and visceral resistance), 5-HT (under pathological stimuli or stress, 5-HT is released by enterochromaffin cells to induce peristalsis reflex), and so forth (36).

In the present study, our data showed that LP-KFY02 helped mice with constipation in restoring these neurotransmitter levels to normal that helped relieve constipation. The KFY02H group showed a more pronounced effect. We considered that favorable intestinal flora state mediated by LP-KFY02 influenced the production of neuroendocrine factors and gastrointestinal hormones through the brain-gut axis, thereby regulating intestinal motility.

3. Probiotics affect the intestinal mucosal immune system.

The intestinal mucosal barrier mainly refers to the digestive tract barrier composed of the constantly renewed intestinal epithelial cells as the structural basis (37). The intestinal mucosal barrier is the first barrier between the intestinal lumen and the internal environment of microorganisms, and its high selective permeability ensures the dynamic balance of the exchange of substances in the internal and external environments of the body (38). Studies showed that patients with FC had increased numbers of CD3 +, CD4 +, CD8 +, and CD25 + T cells, accompanied by lymphocyte proliferation and increased intestinal permeability, indicating that these patients had the activation of the immune system and inflammation of the intestinal mucosa reaction (39). Other studies showed that patients with constipation had an increased number of intestinal mucosal immune cells, such as mast cells and lymphocytes (40). Activated immune cells release many inflammatory cytokines and neurotransmitters, which in turn disrupt the intestinal mucosal barrier and trigger abnormalities in intestinal sensation and motility (26). At the same time, the intestinal bacterial imbalance can activate the mucosal immune response, promote the release of inflammatory cytokines, and aggravate the destruction of the intestinal mucosal barrier (26). Claudin is the most important indicator for maintaining the intestinal mucosal mechanical barrier (41). C-kit is mainly expressed on the interstitial cells of the Cajal (ICC) surface, and its main function is to regulate cell proliferation and differentiation. ICC plays an important role in the propagation of gastrointestinal electrical activity and the signal transduction of gastrointestinal neurotransmitters (42). A series of signal transduction pathways can be induced after the specific binding of SCF to c-kit. The SCF/c-kit signaling pathway is related to the depletion of ICCs, and the morphological and structural damage of ICCs in colon tissue leads to a decrease in the expression levels of SCF and c-kit (43). TRPV1 is widely distributed in the sensory neurons, mucosal layer, submucosa layer, and muscular layer of the intestine. It is involved in the regulation of intestinal movement, secretion, and visceral pain sensation (44). GDNF in the gastrointestinal tract is mainly distributed in the muscularis mucosa. Its expression level in colon tissue is significantly higher than that in other gastrointestinal tract tissues, which has an important impact on the distribution of nerves in the colonic muscularis and the function of ganglion cells. GDNF can protect the function of the intestinal mucosal barrier by promoting the proliferation and maturation of intestinal epithelial cells, inhibiting the apoptosis of intestinal epithelial cells, and strengthening the junction between intestinal epithelial cells (45).

Our results showed that LP-KFY02 enhanced the intestinal mucosal barrier function in various ways: (1) After LP-KFY0 treatment the mRNA expression of claudin-1 and occludin were significantly upregulated, thereby regulating tight junctions between intestinal epithelial cells, (2) Through histopathological sections we observed that LP-KFY02 maintained the integrity of gut structure, which avoided constipation induced intestinal barrier damage and intestinal inflammation. (3) Our results also showed that after LP-KFY02 treatment, mRNA expression of SCF, c-Kit, GDNF were significantly upregulated and TRPV1 expression was downregulated significantly (*p* < 0.05), especially in the KFY02H group, which was similar to that in the normal groups. The expression of these genes is associated with the maintenance of intestinal mucosal barrier function and the regulation of intestinal motility. So we considered that favorable intestinal flora environment mediated by LP-KFY02 avoided the activation of mucosal immune responses and the release of inflammatory cytokines, thereby avoiding intestinal sensory and motor abnormalities caused by impaired intestinal mucosal barrier.

## Conclusion

In summary, we studied amelioration effect of LP-KFY02 on low-fiber diet-induced constipation in mice. After LP-KFY02 treatment with constipation mice, the mice fecal water content, intestinal transit ability and defecation time of constipated mice were improved. The mice fecal flora diversity, abundance and structure of the intestinal flora were regulated to the balanced state. The mice serum levels of gut motility related neuroendocrine factors have been increased, the intestinal mucosal barrier function and gut motility related gene expression were regulated in mice colon tissues. At the same time, the mice colon tissue damage were improved. These parameters in the KFY02H group were close to the normal group. These results suggested that LP-KFY02 could be considered as a potential probiotic to help alleviate low-fiber diet-induced constipation. They also provided a theoretical basis for the study of probiotics to relieve constipation by regulating intestinal flora.

## Data availability statement

The original contributions presented in this study are included in the article/supplementary material, further inquiries can be directed to the corresponding author/s.

## Ethics statement

This animal study was reviewed and approved by the Ethics Committee of Chongqing Collaborative Innovation Center for Functional Food.

## Author contributions

RY and XZ performed the majority of the experiments and wrote the manuscript. TL and RX contributed to the data analysis. ZY designed and supervised the study and checked the final manuscript. All authors read and approved the final manuscript.

## Footnotes

1
www.majorbio.com
